# Optimal Treatment for Patients With Cavernous Transformation of the Portal Vein

**DOI:** 10.3389/fmed.2022.853138

**Published:** 2022-03-24

**Authors:** Bo Wei, Zhiyin Huang, Chengwei Tang

**Affiliations:** ^1^Department of Gastroenterology, West China Hospital, Sichuan University, Chengdu, China; ^2^Laboratory of Gastroenterology and Hepatology, West China Hospital, Sichuan University, Chengdu, China

**Keywords:** cavernous transformation of the portal vein, meso-Rex bypass, portal vein recanalization, splenorenal shunt, TIPS

## Abstract

Cavernous transformation of the portal vein (CTPV) is a sequela of extrahepatic and/or intrahepatic portal vein obstruction caused by a combination of local and risk factors. It was ever taken as a relatively rare disease due to its scant literature, which was mainly based on clinical series and case reports. CTPV often manifests as gastroesophageal variceal bleeding, splenomegaly, and portal biliopathy after the long-term insidious presentation. It is unable for CTPV to be recanalized with anticoagulation because it is a complete obstruction of the mesentericoportal axis. Endoscopic therapy is mainly used for temporary hemostasis in acute variceal bleeding. Meso-Rex shunting characterized by portal-flow-preserving shunts has been widely performed in children with CTPV. The multitude of complications associated with CTPV in adults can be effectively addressed by various interventional vascular therapies. With the ubiquity of radiological examinations, optimal treatment for patients with CTPV becomes important. Multivisceral transplantation, such as liver-small intestinal transplantation, may be lifesaving and should be considered for patients with diffuse mesenteric venous thrombosis.

## Introduction

Cavernous transformation of the portal vein (CTPV) is usually secondary to long-standing portal vein thrombosis (PVT) or portal vein obstruction. As a sequela of portal vein obstruction, especially complete extrahepatic portal vein obstruction (EHPVO), fibroblasts transform the clot into a firm, collagenous plug in, which tortuous venous channels develop. On this basis, portal hypertension caused by PVT may promote the formation of periportal or intrahepatic venous collateral circulation, resulting in CTPV, bypassing extrahepatic portal venous occlusion over time, and interrupting portal inflow (1–5). CTPV may occur as early as 6–20 days after EHPVO, with an average time of approximately 5 weeks (3, 5, 6). It was first reported in 1869 by Balfour and Stewart and is also referred to as a portal cavernoma due to the sponge-like appearance of the portal vein (7).

Cavernous transformation of the portal vein may remain insidious for long-term presentation. It often manifests as gastroesophageal variceal bleeding, splenomegaly, and thrombocytopenia. The biliary tree may undergo morphological and functional changes due to CTPV, resulting in obstructive jaundice. It has recently been termed “portal biliopathy” (8). The mortality rate due to variceal hemorrhage is 5%, and the overall mortality rate is 10% in both adults and children with CTPV (9, 10). Even worse, more than 43% of obstetric patients with non-cirrhotic prehepatic portal hypertension and the development of esophageal varices will suffer significant variceal bleeding during pregnancy. This potentially catastrophic complication is associated with a 33% perinatal mortality rate (11). Recently, it was suggested that the presence of ascites may be of great importance to predict the incidence of death in patients with CTPV, mainly attributed to its close correlation with the deterioration of liver function (12–14). CTPV and chronic PVT remain a challenge at the time of transplant. They are relative contraindications to liver transplantation at many centers because the non-physiological portal flow may increase perioperative and postoperative risks associated with surgical techniques.

## Etiology

**Figure 1:**
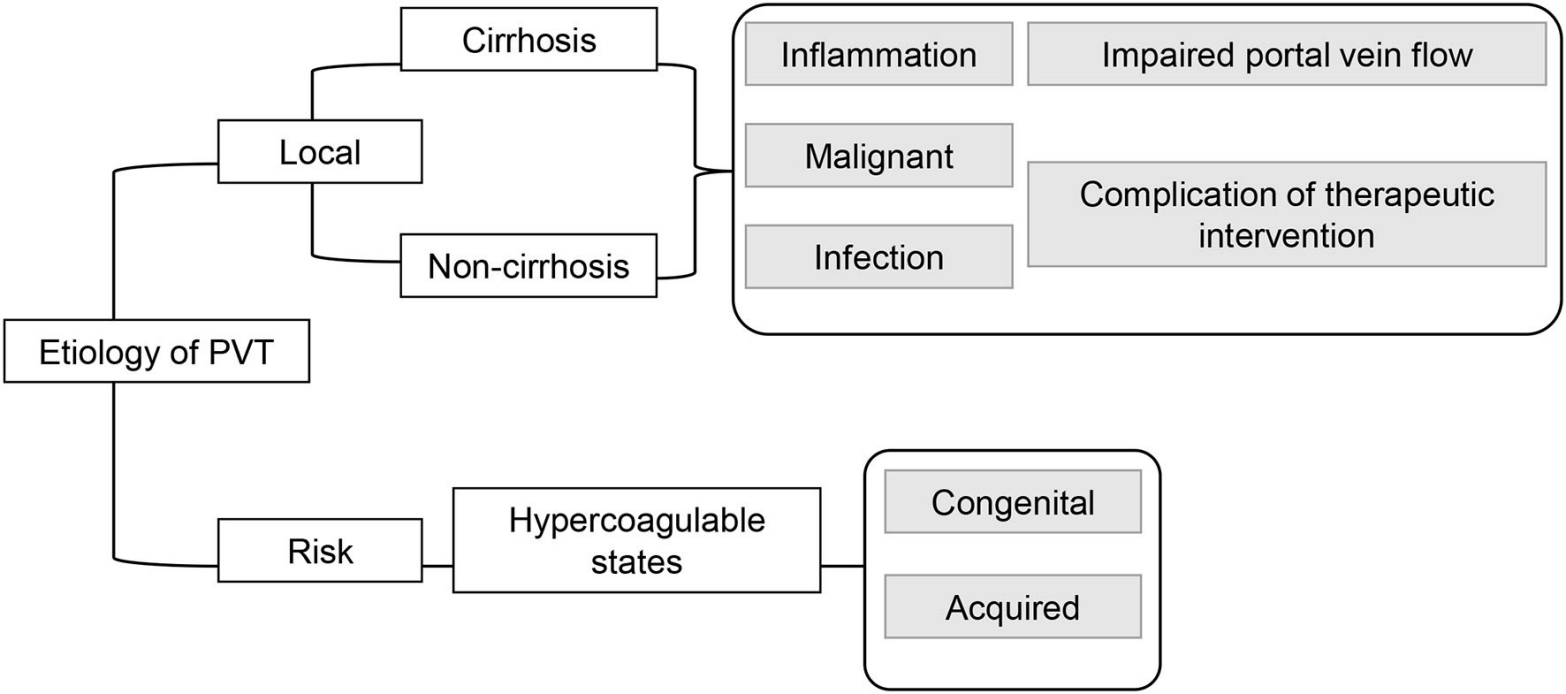
The major causes of portal vein thrombosis (PVT).

The hemodynamics of the portal venous system are characterized by low pressure, slow flow, and high volume. Similar to other venous thromboses, the formation of PVT is also multifactorial due to reduced blood flow, endothelial injury, and hypercoagulability. PVT is caused by a combination of local and risk factors (Figure 1).

The causes of CTPV and acute PVT in adults are similar. The commonly affected population may be divided into cirrhotic and non-cirrhotic patients. The composition ratio of cirrhotic and non-cirrhotic cases varies by region. A study of 254 autopsies from Sweden reported that 28% of PVT cases were cirrhotic, whereas non-cirrhotic PVT accounted for most of the rest (15). PVT occurs in ~20% of cirrhotic patients, who are a great body of inpatients in China. The development of cirrhotic PVT is closely associated with static portal blood flow due to portal hypertension and endothelial injury due to intestinal infection and therapeutic inflammation. In non-cirrhotic patients, a systemic hypercoagulable state is often implicated in PVT (Figure 1). The occurrence of vascular malformation in children suggests that a congenital defect is often a contributing factor, such as prior umbilical cannulation and infection. Inherited and acquired prothrombotic disorders (e.g., latent myeloproliferative disorder, protein C or protein S deficiency, and antiphospholipid syndrome) and thrombotic stimuli, such as pregnancy or oral contraceptives, intra-abdominal infection, and surgical procedures, have been observed in adults, while repeated abdominal infections, sepsis, abdominal invasive procedures, trauma, and congenital anomalies, with or without a prothrombotic state, have been alleged to lead to CTPV in children (16–19). After the exclusion of the aforementioned causes, the etiology of EHPVO remains obscure in up to 50% of patients (20).

## Prevalence

The prevalence of PVT in the general population ranges from 0.7 to 1/10^5^ (21). It is increased ~1,000-fold in cirrhotic patients, with a range of 0.6%−50%, increasing proportionally with liver cirrhosis severity (22, 23). Epidemiological data of non-cirrhotic PVT in the general population are limited by its infrequency. The prevalence of EHPVO was estimated to be as high as 1.0% in an autopsy study in Sweden (15). However, it was much lower (3.7 per 100,000 population) in another Swedish study based on hospital discharge diagnoses (21), suggesting that EHPVO commonly develops at a late stage of some diseases. CTPV among adults is quite rare, with an incidence of 15.6% among EHPVO (24).

The concept of CTPV as a relatively rare disease is mainly based on clinical series and case reports. The literature on CTPV is scant. Therefore, there is considerable heterogeneity in its treatment. Accordingly, the current therapies are mainly extrapolated from cohort studies and/or based on clinician expertise. Optimal treatment for patients with CTPV becomes important with increasing identification by imaging modalities.

## Anticoagulation

Anticoagulation is the cornerstone of treatment for acute PVT without malignancy and should be initiated at diagnosis. Anticoagulant treatment must be considered in cirrhotic patients with PVT following the implementation of adequate prophylaxis for gastrointestinal bleeding (25). However, the main portal vein of CTPV is commonly considered to be unable to be recanalized with anticoagulation because it is a complete obstruction of the mesentericoportal axis. There is only one case reported in which the portal cavernoma was reversed by long-term (5 years) anticoagulation (26). Anticoagulation may lessen the degree of bile duct obstruction in certain patients with CTPV with cholangiopathy, probably by maintaining the patency of periportal or intrahepatic venous collaterals and reducing the compression of the bile duct (27). It is not beneficial for children to take long-term anticoagulation since the procoagulant state is an occasional cause of chronic non-cirrhotic EHPVO (28, 29).

## Endoscopic Management

Endoscopic therapy cannot reduce portal hypertension and is mainly used for temporary hemostasis in acute variceal bleeding. Endoscopic variceal ligation (EVL) is recommended for the management of active esophageal variceal bleeding (12, 30, 31). When acute bleeding from isolated gastric varices and gastroesophageal varices type 2 (GOV2) extends beyond the cardia, sclerotherapy or endoscopic therapy with tissue adhesive should be taken into account. Either ligation or endoscopic therapy tissue adhesive can be used for GOV1 bleeding. Primary prophylaxis with endoscopic treatment has been recommended for patients with cirrhosis with EHPVO by the Baveno VI consensus (31). Recently, endoscopic ultrasound-guided coil (EUS-coil) therapy has emerged as a promising option due to its therapeutic superiority over endoscopic glue injection. It is believed that EUS-coil therapy is the next intervention for primary and secondary hemostases of gastric variceal bleeding and will result in a paradigm shift (32, 33).

Multiple endoscopic procedures may become a local risk for PVT due to traumatic inflammation in the portal system. When portal-systemic collaterals are blocked by endoscopic procedures without being diverted, the increased portal pressure carries an increased risk of biliary complications, mostly from the suppression of periportal collateral vessels. Thus, portal decompression should be performed early, even if the risk of bleeding is not high (34–37).

## Surgical Shunting

Gastroesophageal devascularization between the portal and azygos veins alone without a shunt has been less performed recently due to its higher rebleeding rate and lower 5-year survival rate (38, 39). There are several surgical approaches by which portacaval shunts can be established to decompress portal hypertension (40–42). The experience from adults indicated that non-selective surgical shunts such as side-to-side splenorenal or meso-caval shunts can totally divert the portal flow toward the systemic circulation but apparently potentiate ischemia-perfusion to the liver at the same time (40, 43). In this regard, non-selective shunts and their derivative techniques are taken into account only in patients with refractory life-threatening bleeding (44–46). Selective shunts, such as the distal splenorenal shunt developed by Warren et al., have been considered as effective as non-selective shunts in controlling variceal bleeding. It preserves a portion of portal perfusion to the liver and is better in preventing portosystemic encephalopathy (40, 47). However, widespread use of distal splenorenal shunts has been limited in terms of technical requirements, particularly in adults (38).

Meso-Rex shunting is a recently established surgical technique. The patient's internal jugular vein is used as an autograft by which the superior mesenteric vein blood flow is diverted into the umbilical portion of the left portal vein. This technique sufficiently restores a substantial portion of physiological portal blood flow to the liver and avoids the long-term adverse consequences of portosystemic shunting, such as liver atrophy and growth retardation (16, 40, 45). This portal-flow-preserving shunt has been widely performed in children with CTPV. The bypass patency rates might reach 100% 1 year after the operation and remained at 78% during the 10-year follow-up. Of the 490 reported cases, only three deaths occurred (Table 1) (40, 45, 48–65).

**Table 1:** Studies of meso-Rex bypass in reported series.

**First author, year**	***n***	**Age (yr.)**	**Inflow**	**Bypass graft**	**Outflow**	**Follow up**	**Bypass patency**	**Death**
Goyet et al. (50)	7	0.6–15	SMV	LIJV, RGEV	LPV	Median, 1.5 y	7	0
Gehrke et al. (52)	13	1.2–14.2	SMV	IJV	UP of LPV	Median, 1y	13	0
Mack et al. (53)	11	N/A	SMV	N/A	LPV	1 y	10	0
Superina et al. (49)	33	0.3–14	SMV	IJV	LPV	1–7 y.	31	0
Mack et al. (54)	8	9.7 ± 1.8	SMV	N/A	LPV	1 y	8	0
Stringer et al. (55)	11	0.9–15	SMV	IJV	UP of LPV	7 m-5 y	10	0
Lautz et al. (56)	45	6.8 ± 4.1	SMV	N/A	LPV	24 m	39	0
Sharif et al. (51)	30	0.4–14.2	SMV	LIJV, UV, GV, LCV, prosthetic material	LPV	Median, 8 y	23	1
Lautz et al. (46)	65	7.0 ± 4.8	SMV	IJV, IMV, CV	LPV	Median, 4.5 y	63	0
Guérin et al. (59)	32	4.0–10.6	SMV, SpV	IJV, PTFE graft	Rex fossa	18–107 m	23	0
R Bhat et al. (61)	65	0.3–20.4	SMV	IJV, CV, IMV, SpV, PTFE graft	LPV	0.07–111 m	56	0
Z Wei et al. (64)	22	4.5–13	SMV	SpV	LPV	12–48 m	20	0
T Lautz et al. (60)	16	2.3–11.3	SMV	N/A	LPV	1 y	14	1
N Tantemsapya et al. (63)	37	1.2–15	SMV	N/A	LPV	1.5–10 y	29	0
Wu et al. (57)	68	5.0 ± 3.0	SMV	LIJV, CV	LPV	Median, 1.7 y	64	0
Tang et al. (58)	13	11–62	SMV or SpV trunk, or confluence of SMV and SpV	RIJV, allogeneic iliac vein	Sagittal part of LPV, recanalized UV	0–67 m	7	0
Brichard et al. (62)	14	22–66	SMV	LIJV, right femoral vein, PTFE graft, or in combination.	Rex recess	2–169 m	9	1
Total	490							3

*CV, coronary vein; GV, gastric vein; IJV, internal jugular vein; IMV, inferior mesenteric vein; LCV, large colic vein; LIJV, left internal jugular vein; LPV, left portal vein; N/A, not available; RGEV, right gastroepiploic vein; RIJV, right internal jugular vein; SMV, superior mesenteric vein; UP, umbilical potion; UV, umbilical vein*.

Later, several modifications of meso-Rex bypass using alternate sources of venous inflow and graft conduits in children when standard meso-Rex shunting cannot be achieved (66, 67). It has been recommended by the Baveno consensus that meso-Rex bypass, the most physiological shunting and the only “curative surgery,” should be performed for patients in the early stage of EHPVO (31, 44, 68, 69). However, the controversy concerning the legitimacy of its utilization in an asymptomatic child is still unresolved. Available data show an inverse correlation between the restoration of appropriate portal flow to the liver following meso-Rex shunt and the age of the patient (49, 69). It has been proposed that the assessment of surgical feasibility should be performed in all children with CTPV in a more prophylactic manner (70–72), while in fact, many medical centers routinely defer preemptive meso-Rex bypass until intractable and refractory symptoms are established.

Inspired by the success of meso-Rex bypass in children with CTPV, a modified meso-Rex bypass (splenic vein to cystic part of the portal vein) was successfully performed in an adult patient after liver transplantation (73). Recently, a novel meso-Rex bypass with umbilical vein recanalization and intraoperative stenting was reported (58). It may reduce the risk of intravascular thrombosis and bypass vessel occlusion compared with traditional meso-Rex shunting. The limited data on surgical shunting in adults with CTPV will encourage more studies for adult patients.

Cavernous transformation of the portal vein was once considered an absolute contraindication for liver transplantation due to unsatisfactory portal flow to the graft (74, 75). However, incremental refinements of the surgical technique have allowed recipients with CTPV to undergo liver transplantation using a prominent collateral vein, dilated coronary vein, interposition graft, portal vein arterialization, or some anastomotic methods, including renoportal or cavoportal anastomosis, for inflow reconstruction. Occasionally, multivisceral transplantation, such as liver-small intestinal transplantation, may be lifesaving and should be considered for patients with diffuse mesenteric venous thrombosis (74, 76–83).

## Interventional Vascular Therapies

Surgical challenges have promoted the application of interventional technology in patients with CTPV. The multitude of complications due to CTPV can be effectively addressed by various interventional vascular therapies.

### Modified Transjugular Intrahepatic Portosystemic Shunt

**Figure 2:**
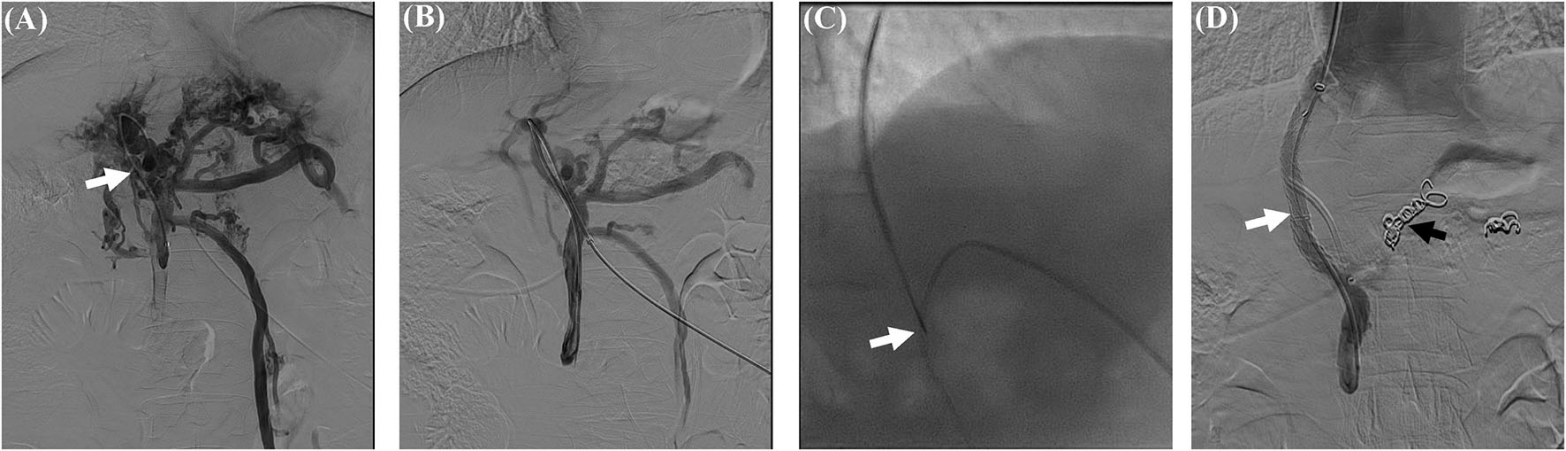
PVR-TIPS creation in a patient with CTPV. 53 years/M, CTPV with cirrhosis. **(A)** Percutaneous transhepatic portography revealed extrahepatic portal obstruction and collateral vein formation (white arrow). **(B)** Percutaneous portography after portal vein recanalization (PVR) by an 8 mm × 60 mm balloon catheter. **(C)** A catheter was placed in the portal vein as a marker for TIPS puncture (white arrow). **(D)** The portosystemic shunt was created with a covered stent (white arrow) after the embolization of the coronary vein with coils (black arrow). CTPV, cavernous transformation of the portal vein; PVR, portal vein recanalization; TIPS, transjugular intrahepatic portosystemic shunt.

Cavernous transformation of the portal vein has previously been considered contraindicated for transjugular intrahepatic portosystemic shunt (TIPS) due to technical difficulties and its vital complications. However, it has been feasible for modified TIPS (mTIPS) creation in some patients with CTPV since 2006 (84). The procedure has been modified and evolved to include transjugular, transhepatic, and transsplenic accesses to assist with portal vein recanalization (Figure 2). Rates of technical success of recanalization have been reported in a range of 75–100% in incomplete occluded portal veins (85). Technical success is associated with the degree of occlusion of the main portal vein. Cavernous transformation may increase the technical difficulty. There are two strategies for intrahepatic portosystemic shunt placement, namely, (1) portal recanalization and conventional implantation of the stent to create intrahepatic portosystemic shunt and (2) insertion of the stent between the hepatic vein and a periportal collateral vessel without portal recanalization for whom recanalization of the portal vein is not possible but there are dilated veins of a cavernous transformation (86–92). In clinical practice, the second strategy is also challenged by the injury of surrounding collaterals (93, 94). Intraparenchymal injection of CO_2_ or wedged hepatic venous portography may be helpful to guide the intraparenchymal needle toward the target intrahepatic portal tree (93, 95). The existence of both a high- and a low-pressure portal venous network in patients with CTPV should be considered. Therefore, mTIPS creation requires careful selection of an intrahepatic portal vein with high pressure to achieve adequate portal decompression and improve clinical success (96). However, it is usually difficult to obtain an accurate hepatic venous pressure gradient (HVPG) in patients with portal hypertension due to the presence of intrahepatic venous-venous shunts (97). Thus, direct measurement of portal pressure and calculation of portosystemic pressure gradient (PPG) are more meaningful for the establishment of portosystemic shunt than HVPG. To reconstruct a physiological hepatopetal flow for patients with severe spontaneous portal-systemic shunts (SPSS), it is necessary to obliterate SPSS, such as balloon-occluded retrograde transvenous obliteration (BRTO), during mTIPS placement (69, 98).

Performance of portal vein recanalization (PVR) or its combination with TIPS was initially aimed at optimizing the transplant candidacy of patients with PVT or CTPV. Currently, the placement of intrahepatic portosystemic shunts has become an alternative treatment for PVT and CTPV, which is not a distinct contraindication to TIPS procedures. It has been considered salvage therapy when endoscopic treatment is unsuccessful in patients with chronic PVT and cavernous transformation (96). Although there are many technical difficulties in creating mTIPS, it is still a major procedure for chronic PVT or CTPV in China due to many cirrhotic patients.

### Portal Vein Stenting

Theoretically, the mTIPS technique focuses on addressing intrahepatic resistance and should be beneficial to cirrhotic CTPV. EHPVO alone is the characteristic of non-cirrhotic patients with CTPV. Insertion of portal vein stent (PVS) alone may alleviate extrahepatic portal hypertension and prevent rebleeding effectively only by recanalization of the obstructed portal vein, venoplasty, and stenting (96). PVS intervention may be more suitable for non-cirrhotic patients than mTIPS because it preserves adequate physiological blood inflow to the liver. However, it is reasonable to be aware of the possibility of catheterizing the portal vein remnant and the patency of the major splanchnic vessels by Doppler ultrasound and CT (99). A new classification for CTPV proposed by Marot et al. was formulated with the aim of selecting which patients could be considered for portal angioplasty. Intrahepatic involvement with extension to the origin of the hepatic segmental branches and distal branches was ultimately associated with technical failure or with early stent thrombosis due to insufficient blood outflow. Therefore, PVR alone should not be considered in these patients (100). Most of the patients (90%) had considerably improved portal hypertension-related symptoms. This procedure is known to be an effective treatment for PV obstruction after liver transplantation and from primary malignancy (101–105). A retrospective study with 42 cases with PV obstruction following non-transplant hepatobiliary or pancreatic surgery considered that portomesenteric venoplasty and stent placement are safe with a high rate of technical success if performed before chronic occlusion (102). The long-term stent patency in patients who underwent PVS varied with different ages, causes, and techniques they accepted (Table 2) (99–103, 105–110).

**Table 2:** Recent studies of portal vein recanalization (PVR)/angioplasty in reported series.

**First author, year**	***n***	**Age, year**	**Cause**	**Technique**	**Follow up, month**	**Stent patency**	**Death**
Semiz-Oysu et al. (106)	19	Mean 36.4 (0.75–79)	N/A	TH, TJ+TH, balloon + stent	2–58	13	5
Jeon et al. (108)	21	Mean 65.6 (26–78)	HPB surgery	TH, balloon + stent	mean 12.5 (1.4–25)	20	0
Kato et al. (103)	29	65.9 ± 10.0 (38–83)	HPB surgery	TH, surgical approach via the ileocolic vein, balloon + stent	19.1 ± 24.9	22	1
Cavalcante et al. (107)	22	Median 2.7 (0.7–11.8)	Liver transplantation	transmesenteric approach via minilaparotomy with or without TH, balloon + stent	Median 88.9 (20.9–159.4)	17	2
Naik et al. (105)	19	Median12 (7–15)	Liver transplantation	TH, TS, balloon	Median 16 (5–35)	18	0
Marot et al. (100)	13	47 ± 12 (22–60)	Unknown (3), inflammation (7), abnormal coagulation factors (5)	TH, balloon + stent	42 ± 28 6–112)	10	2
Kim et al. (110)	31	Mean 52 (25–62)	Liver transplantation	TH, balloon + stent	Median 54.2 (0.5–192.4)	26	2
Mugu et al. (102)	38	60.1 ± 11.3 (22.3–78.3)	HPB surgery	TH, TS, balloon + stent	8.6 ± 8.8	29	6
Lee et al. (101)	60	62.5 ± 13.7 (18–88)	HPB surgery	TH, balloon + stent	20.8 ± 24 (0–101.5)	47	14

*TH, transhepatic; TJ, transjugular; TS, transsplenic; N/A, not available; HPB, hepatobiliary or pancreatic*.

**Figure 3:**
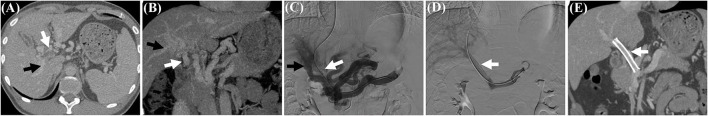
PVS in a patient with CTPV. 35 years/M, CTPV without cirrhosis. **(A)** and **(B)** CT images and reconstruction revealed occlusion of main portal vein and cavernoma (white arrow) and visible branches of th portal vein (black arrow). **(C)** Transjugular portography showed complete occlusion of the portal vein (white arrow) and collateral vein which is not directly connected with intrahepatic portal branches (black arrow). **(D)** An 8 mm × 60 mm bare stent was inserted into the main portal vein (white arrow), and the intrahepatic portal vein was clearly shown by portography. **(E)** CT reconstruction showed a patent stent (white arrow) in the main portal vein 1 year after the procedure. PVS, portal vein stenting; CTPV, cavernous transformation of the portal vein.

Similar to mTIPS, several routes for gaining access to the portal system are emerging in PVS intervention, even though the portal vein has been obliterated and becomes a fibrotic cord. These multimodality cutting-edge therapeutic approaches, which encompass transjugular, transhepatic-intrahepatic portal vein branch using US guidance, transsplenic-US guidance, trans-ileocolic-mini-laparotomy (i.e., hybrid approach), as well as the trans-recanalized paraumbilical vein either alone or in combination, push the development of PVS (Figure 3) (93, 99, 111–115). Transhepatic-intrahepatic portal vein branch using US guidance has been by far the most common way of gaining access to perform PVS (116). Direct access to the left portal vein risks Rex's recess (116); this option should consequently be weighed before meso-Rex bypass. For patients with atresia of the portal vein, which cannot be recanalized by the endovascular (the main portal vein) approach, a stent shunt between the intrahepatic portal branch and the large collateral vessel should be taken into consideration (96). Currently, there is no clear evidence supporting the use of either of these approaches over the others. It is also worth noting that the preservation of the splenic-mesenteric confluence and the left portal vein should be considered mandatory for patients to avoid compromising the possibility of meso-Rex shunting and liver transplantation (99). Moreover, PVS in non-cirrhotic CTPV may avoid the common complications related to mTIPS, including chronic recurrent encephalopathy, liver failure, and congestive cardiac failure (93).

### Treatment of Portal Cavernoma Biliopathy

Extrinsic compression of the bile duct by collaterals and/or ischemic damage due to altered biliary vascularization has been implicated in the pathogenesis of portal cavernoma biliopathy (117). Invasive treatment strategies of portal cholangiopathy should only be performed in patients with clinical symptoms (accounting for ~5–38% of abnormalities seen by magnetic resonance cholangiopancreatography) in view of high bleeding risk, even in the presence of obvious stenosis on imaging (28, 117, 118).

Definitive management usually requires combined approaches aimed at treating both portal hypertension and biliary stenosis, including endoscopic bile duct dilation/stenting, stone extraction, cholecystectomy, bilioenteric anastomosis, and the portal decompression strategies mentioned above (117, 119, 120). In our previous study, we successfully treated a patient with CTPV with intractable biliary obstruction following TIPS placement using a novel magnet-assisted endoscopic biliary-duodenal anastomosis (121), which seems to be a promising solution for portal biliopathy under certain conditions, particularly in patients with a perceived increased risk of conventional bilioenteric anastomosis.

## Conclusion

Patients with CTPV should be treated in regional central hospital with strong surgical and interventional teams. Meso-Rex bypass is recommended for children with CTPV. Incremental refinements of the interventional techniques, such as mTIPS creation and PVS, can greatly benefit adult patients with CTPV by reducing the need for liver transplantation or transforming contraindications of liver transplantation into indications. The optimal treatment for patients is a personal interventional strategy on the basis of their heterogeneity in mesentericoportal obstruction.

## Author Contributions

CT contributed to conception and design of the study. ZH analyzed the literature and drew the tables. BW wrote the first draft of the manuscript. All authors contributed to manuscript revision, read, and approved the submitted version.

## Funding

This study was supported by the National Natural Science Fund of China (82170625, U1702281), the National Key R&D Program of China (2017YFA0205404), and the 135 projects for disciplines of excellence of West China Hospital, Sichuan University (ZYGD18004).

## Conflict of Interest

The authors declare that the research was conducted in the absence of any commercial or financial relationships that could be construed as a potential conflict of interest.

## Publisher's Note

All claims expressed in this article are solely those of the authors and do not necessarily represent those of their affiliated organizations, or those of the publisher, the editors and the reviewers. Any product that may be evaluated in this article, or claim that may be made by its manufacturer, is not guaranteed or endorsed by the publisher.
